# Admixture mapping and selection scans identify genomic regions associated with stomatal patterning and disease resistance in hybrid poplars

**DOI:** 10.1002/ece3.10579

**Published:** 2023-10-24

**Authors:** Karl C. Fetter, Stephen R. Keller

**Affiliations:** ^1^ Department of Plant Biology University of Vermont Burlington Vermont USA; ^2^ Department of Ecology and Evolutionary Biology University of Connecticut Storrs Connecticut USA

**Keywords:** admixture mapping, disease ecology, hybrids, *Melampsora*, *Populus*, quantitative genetics, stomata, trade‐offs

## Abstract

Variation in fitness components can be linked in some cases to variation in key traits. Metric traits that lie at the intersection of development, defense, and ecological interactions may be expected to experience environmental selection, informing our understanding of evolutionary and ecological processes. Here, we use quantitative genetic and population genomic methods to investigate disease dynamics in hybrid and non‐hybrid populations. We focus our investigation on morphological and ecophysiological traits which inform our understanding of physiology, growth, and defense against a pathogen. In particular, we investigate stomata, microscopic pores on the surface of a leaf that regulate gas exchange during photosynthesis and are sites of entry for various plant pathogens. Stomatal patterning traits were highly predictive of disease risk. Admixture mapping identified a polygenic basis of disease resistance. Candidate genes for stomatal and disease resistance map to the same genomic regions and experienced positive selection. Genes with functions to guard cell homeostasis, the plant immune system, components of constitutive defenses, and growth‐related transcription factors were identified. Our results indicate positive selection acted on candidate genes for stomatal patterning and disease resistance, potentially acting in concert to structure their variation in naturally formed backcrossing hybrid populations.

## Graphical abstract

Hybrid poplars with increased stomatal density on the upper leaf surface have higher disease and lower growth. Quantitative genetic and admixture mapping unravels aspects of disease ecology, stomatal morphology, and ecophysiology in advanced generation hybrids.

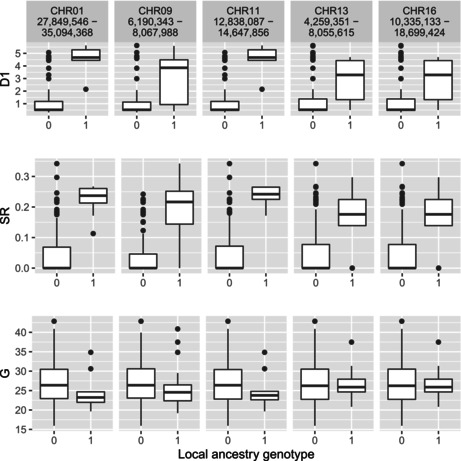

## 1 INTRODUCTION

The evolutionary and ecological consequences of hybridization across landscapes have received considerable attention in plants, particularly in sunflowers (Rieseberg et al., 2007), monkeyflowers (Chase et al., 2017), and poplars (Suarez‐Gonzalez et al., 2018). Hybridization studies in poplars have been influential in developing our understanding of how admixture between foundational tree species can alter biotic interactions between herbivores (Whitham et al., 1996), fungi (Bailey et al., 2005), and entire ecosystems (Bailey et al., 2009). Underlying ecological change in hybrid populations are the novel phenotypes expressed as a result of gene flow between isolated genomes, heterosis, and transgressive segregation, among other phenomena.

Research from poplar hybrid zones indicates that their genomes are porous to the movement of genetic variants (Bailey et al., 2009). Multiple mechanisms of evolutionary change have been studied in poplar hybrid zones, in particular adaptive introgression, when a gene evolved in one species is introduced to another via reproduction and subsequently experiences selection to maintain its function (Rieseberg & Carney, 1998). However, less attention has been paid to the maladaptation of hybrids and the phenotypic and genomic effects of hybridization and backcrossing in natural systems. Plant diseases and pests have been indicated as important players structuring ecological systems (Floate et al., 2016). Hybrids, while often thought of as “super genotypes” in plant breeding, are susceptible to decreased fitness as a result of trade‐offs and mismatches of growth–defense syndromes that have evolved in different evolutionary and ecological contexts (Fetter et al., 2021). Advanced generation hybrids offer a useful opportunity to employ methods to identify trade‐offs and to use genomic methods to categorize the genes associated with ecologically relevant traits.

A major goal of ecological genomics is to link phenotypic and genomic variation to find genes underlying ecological processes. Advances in sequencing technology have enabled the discovery of genetic polymorphisms covering a large fraction of a genome. While many association genetic methods have been developed to help control for false positives arising from population structure within a species, a sample generated from hybrids is a considerably more difficult problem (Shriner et al., 2011), due to large blocks of loci in high LD as a result of relatively few recombination events in hybrids. Association genetic methods based on allele frequency variation are likely to yield many false positives in hybrid populations. Admixture mapping was invented to enable association genetics in hybrid or admixed populations (Smith & O'Brien, 2005). Rather than using the identity of a base pair at a locus (i.e., A, G, T, or C), a locus is represented as homozygous or heterozygous for ancestry from either parental species/population. Thus, the phenotypic associations are made to the local ancestry of a locus (Shriner et al., 2011).

*Populus* is a genus of long‐lived, wind‐pollinated trees which exhibit remarkable ecological amplitude. Species in the genus occur beyond the tundra‐boreal forest ecotone in northern Alaska and Canada (e.g., *P. balsamifera*, Breen, 2014). Poplars extend to the humid subtropical forests of the southeastern American coastal plain (e.g., *P. heterophylla*), and to the arid deserts of the Middle East (e.g., *P. euphratica*). *Populus* species will readily form fertile hybrids, and bi‐directional crossing is frequent (Suarez‐Gonzalez et al., 2018), although not all species can do this (e.g., *P. balsamifera* × *deltoides* hybrids will only cross into *P. balsamifera*, Thompson et al., 2010). Given the promiscuity of poplars and their ability to persist as clones on the landscape, hybrid backcrosses are expected to occur, creating the biological conditions to employ admixture mapping.

In a previous study, we found evidence of hybrids expressing a genetic correlation between stomatal traits and resistance to the basidiomycete leaf rust, *Melampsora medusae* (Fetter et al., 2021). Increases in upper stomatal density correspond to a loss of resistance, particularly when a larger fraction of the stomatal pore area was shifted to the upper surface. Stomatal morphologies of parental species contributing hybrids indicated that they evolved in response to selection for contrasting growth–defense syndromes. The increased phenotypic variance of hybrids allowed for maladapted phenotypes to be expressed, and identified. The genomic architecture of the growth–defense trade‐offs mediated by the contrasting stomatal morphologies remains unknown, in particular, which modes of selection predominate in candidate genes involved in the trade‐off. Here, we explore the phenotypic and genomic relationships between disease, stomatal, and ecophysiological traits. Focusing on a pair of species, we use quantitative genetic, and association genetic models to identify trade‐offs and their genomic architecture. Finally, we test whether candidate associations show evidence of positive selection that is potentially driven by pathogen‐induced selection.

## 2 MATERIALS AND METHODS

### 2.1 Plant collections, phenotypes, and sequencing

Plant material from 534 individuals was collected in 2013 and 2014 in Canada and the United States. Cuttings were stored in a cold room until they propagated in containers and planted in a common garden in Vermont, USA in June 2014. After overwintering, ecophysiology, height growth, and bud phenology traits were measured (Table 1). For a detailed explanation of phenotypes, see Fetter et al. (2021). Following genotyping and genetic analysis, several hybrid individuals were identified, including hybrids from crosses with *P. trichocarpa*, *P. angustifolia*, and *P. deltoides* (Chhatre et al., 2018; Fetter et al., 2021).

**TABLE 1:** Trait definitions, abbreviations, and units.

Definition	Abbreviations	Units
Disease		
Disease severity scale 1 (2015)	D1	Ordinal
Disease resistance scale 1 (2015)	R1	Ordinal
Disease severity scale 1 (2016)	D2	Ordinal
Disease severity scale 2 (2016)	D3	Ordinal
Disease presence/absence (2016)	dis_pres	Binary
Stomatal patterning		
Stomatal ratio	SR	None
Pore length ratio	LR	None
Stomatal cover ratio	*f*_s_R	None
Upper (adaxial) stomatal density	SD_AD	mm^2^
Lower (abaxial) stomata density	SD_AB	mm^2^
Total stomatal density	D	mm^2^
Upper (adaxial) pore length	PL_AD	μm
Lower (abaxial) pore length	PL_AB	μm
Upper (adaxial) stomatal cover	$f_{S_{U}}$	None
Lower (abaxial) stomatal cover	$f_{S_{L}}$	None
Total stomatal cover	*f*_*S*_	None
Ecophysiology		
Relative growth rate	G	cm
Carbon:Nitrogen	C:N	None
Leaf percent carbon	%C	%
Leaf percent nitrogen	%N	%
Carbon isotope discrimination	Δ^13^C	‰
Nitrogen isotope value	δ^15^N	‰
Specific leaf area	SLA	mm^2^ mg^−1^
Chlorophyll content index	CCI	None
Cumulative growing degree days to bud flush	cGDD‐15	Days
Cumulative growing degree days to bud flush	cGDD‐16	Days

*Melampsora medusae* is a foliar pathogen with two obligate hosts: a poplar as the telial host and a larch (*Larix*) as the aecial host (Feau et al., 2007). A large larch tree was located roughly 50 m from the eastern edge of the plot. Disease severity to *M. medusae* was measured in 2015 using the ordinal scale of Mantia et al. (2013), and in 2016, using both the ordinal scales of La Mantia et al. (2013) and Dowkiw and Bastien (2004) (Table 1).

Genomic DNA was extracted with DNeasy 96 Plant Mini Kits (Qiagen), and libraries were prepared for genotyping by sequencing (GBS). Sequence reads were obtained from an Illumina HiSeq 2500 to generate 100‐bp single‐end reads. Reads were mapped to the *P. trichocarpa* reference assembly version 3.0 (Tuskan et al., 2006), and SNPs were obtained using a modified Tassel pipeline (Glaubitz et al., 2014). SNPs with a minor allele frequency < 0.001 were removed, and only biallelic sites were retained. Sites with a mean depth < 5, genotype quality > 90, and indels were removed. Missing data were imputed with Beagle v5.0 (Browning et al., 2018), and sites with post‐imputation genotype probability <90 and sites with any missingness were removed. A total of 227,607 SNPs were called. Sequence reads are available at the SRA (SRX1605454‐68).

### 2.2 Trait modeling

Best linear unbiased predictors (BLUPs) were fit for each trait from data collected from ramets. Each model included the garden row and column position as fixed effects and individual code as a random effect. Best linear unbiased predictors from 117 individuals, including 83 unadmixed and 34 admixed individuals, were used for regressions. To identify traits that predicted disease presence or absence, a logistic model was fit in (R Core Team, 2021) using the glm function. Disease severity in 2016 (D2) was converted to a binary presence/absence trait by calling BLUPs greater than zero as disease presence. D2 was preferred to other disease traits, as its distribution more clearly lent itself to binary factorization (Figure S1). Regression coefficients were standardized by dividing them by two standard deviations (sensu Gelman, 2008) before plotting.

After evaluating the logistic model, several traits suggested trade‐offs with disease resistance. We fit random slopes and intercepts models from BLUPs with disease resistance (from D1) as the response, and relative growth rate, stomatal ratio, log‐stomatal density as predictors in separate models. A model with stomatal ratio (response) and total stomatal density (predictor) was also fit. Admixture status was included in the model as a grouping effect. Random slope and intercept models were fit with brms (Bürkner, 2017).

### 2.3 Admixture mapping

Admixture mapping requires phased reference haplotypes from unadmixed parental populations to estimate locus‐specific ancestry in a test population. To choose reference individuals for *P. balsamifera*, we estimated global ancestry using ADMIXTURE (Alexander et al., 2009) from the 534 individuals we sequenced. We chose 25 individuals from the western populations of Duck Mountain, Manitoba (DCK), and Hudson Bay, Ontario (HBY), that exhibited minimal signs of admixture with other *Populus* species (i.e., ADMIXTURE q‐matrix > 0.95 *P. balsamifera* at *K* = 2; Figure S2). For the *P. trichocarpa* reference set, 25 individuals with whole‐genome sequences publicly available were chosen from western Washington which were known to lack admixture with *P. balsamifera* (Evans et al., 2014). Whole‐genome sequences were downloaded from https://phytozome.jgi.doe.gov/. In total, 167 individuals were used to develop the locus‐specific ancestry genomic polymorphism data. The test population used in the admixture mapping mixed models contained 117 genes from 276 ramets sampled from 21 populations across the Canadian prairie provinces and western and mid‐western states (Figure S1, Table S1). Individuals from this region were favored for admixture mapping, as gene flow naturally occurs among these accessions that is likely subjected to patterns of selection, mutation, and drift ideal for the mixed models we employed.

The locus positions of the test vcf files were used to filter the *P. trichocarpa* reference set, and after discarding flipped and multi‐allelic sites, 74,878 homologous sites remained. Haplotypes of the reference populations were jointly estimated with fastPHASE (Scheet & Stephens, 2006), setting the following parameters: 20 random starts; 45 EM iterations per run; 5000 samples of the posterior haplotype distribution; the K‐selection function was limited between 5 and 30, at 5 unit intervals; and loci with genotype probability <90 were flagged. Locus‐specific ancestry was determined for the test set via recombination ancestry switchpoint probability estimation with the program RASPberry (Wegmann et al., 2011). The input data sets for RASPberry were as follows: a test set of 485 individuals with 227,607 SNP loci and no site‐wise missingness; two reference populations of 25 individuals each with 74,878 SNP loci; and the ADMIXTURE q‐matrix at *K* = 2. The default recombination rate of 5 cM was used, and population recombination rates set to 120 and 173 for *P. balsamifera* and *P. trichocarpa*. The mutation rate was set to 0.0079365, and miscopy rate to 0.01.

Mixed effects models were fit to identify the association between 24 phenotypes (Table 1) and local ancestry genotypes corrected by the global ancestry of the individual using the Bayesian mixed model (BMIX) of Shriner et al. (2011):

$$f\left(y_{i}\right)=\beta_{0}+\beta_{1}A_{\mathrm{ij}}+\beta_{2}{\bar{A}}_{i}+\varepsilon_{i}$$

where *y*_*i*_ is a vector of BLUPs, *β*_*N*_ are regression coefficients to estimate, *A*_*ij*_ is a vector of local ancestries for the *j* ^th^ locus, ${\bar{A}}_{i}$ is a vector of global ancestries for the *i* ^th^ individual, and $\varepsilon_{i}$ is error variance for the *i* ^th^ individual. Global ancestry was again estimated for each individual with the RASPberry data by summing the frequency of the local ancestries of the homozygous and half the heterozygous genotypes corresponding to the *P. balsamifera* allele. Association probabilities were estimated from chi‐squared test statistics of converted model *p*‐values. The significance level for true associations was set to α/admixture burden (.05/237.5). The admixture burden is an estimate of the number of independent recombining chromosomal tracts in a sample. Admixture burden was estimated from the first‐order autoregressive (AR(1)) models for each locus summed across the genome for all individuals using the function ar from the stats package in R. Manhattan plots were used to visualize *p*‐values of tests across the genome for each trait. Intersections between candidate gene lists were identified with UpSetR (Conway et al., 2017).

### 2.4 Selection scans

Patterns of positive selection were identified with RAiSD (Alachiotis & Pavlidis, 2018) using a composite statistic of the product of *μ* ^SFS^ which measured shifts in the site frequency spectrum, *μ* ^LD^ measuring linkage disequilibrium, and *μ* ^VAR^ which measured genetic polymorphism. The composite statistic is simply named the *μ* in RAiSD terminology. These statistics were calculated in overlapping 50 SNP sliding windows. RAiSD is ideal for identifying hard sweeps, but false positives can be generated by population bottlenecks, background selection, and population structure (Alachiotis & Pavlidis, 2018). As a result, we sub‐sampled the *P. balsamifera* data set to a single deme in the western core which was identified by running ADMIXTURE (Alexander et al., 2009) on all 534 *P. balsamifera* samples from *K* = 2 until resolution of known demes (sensu Keller et al., 2010) within *P. balsamifera* was possible at *K* = 7 (Figure S2). Individuals > 0.98 ancestry in the western core deme at *K* = 7 were selected. The hybrid set was selected from individuals with RASPberry global ancestry < 0.99. RAiSD was run separately for the *P. trichocarpa* reference individuals (*N* = 46), Western core *P. balsamifera* (*N* = 81), and hybrids (*N* = 39; Table S4). The input sequence data set contained 31,545 SNPs common to both *P. trichocarpa* and *P. balsamiera* with no missingness from 165 individuals. We considered windows in the top 1% of *μ* statistics as evidence of a hard selective sweep.

### 2.5 Gene annotation and candidate gene filtering

Gene annotations for the 227,607 SNPs were downloaded from the *P. trichocarpa* v3.0 genome and used to annotate significant loci. After the top 1% of *μ* statistic windows were identified, the overlap of BMIX candidate genes and selection windows was determined using GenomicRanges (Lawrence et al., 2013). Local ancestry sites from genes that passed the BMIX/selection filter were identified, and monomorphic sites removed. With the remaining polymorphic local ancestry sites, boxplots comparing local ancestry genotype and SR, D1, and G were made to evaluate distributions for signs of false positives. Sites that passed this final filter were mapped to *P. trichocarpa* gene annotations and the genes were manually investigated for gene function using www.popgenie.org (Sjödin et al., 2009; Sundell et al., 2015), atgenie.org (Sundell et al., 2015), and TAIR (Berardini et al., 2015). *Populus* gene orthologies to *Arabidopsis* were determined by the best BLAST hit on www.popgenie.org or via manually BLAST on The Arabidopsis Information Resource (www.arabidopsis.org).

## 3 RESULTS

### 3.1 Global and local ancestry of hybrids

**FIGURE 1:**
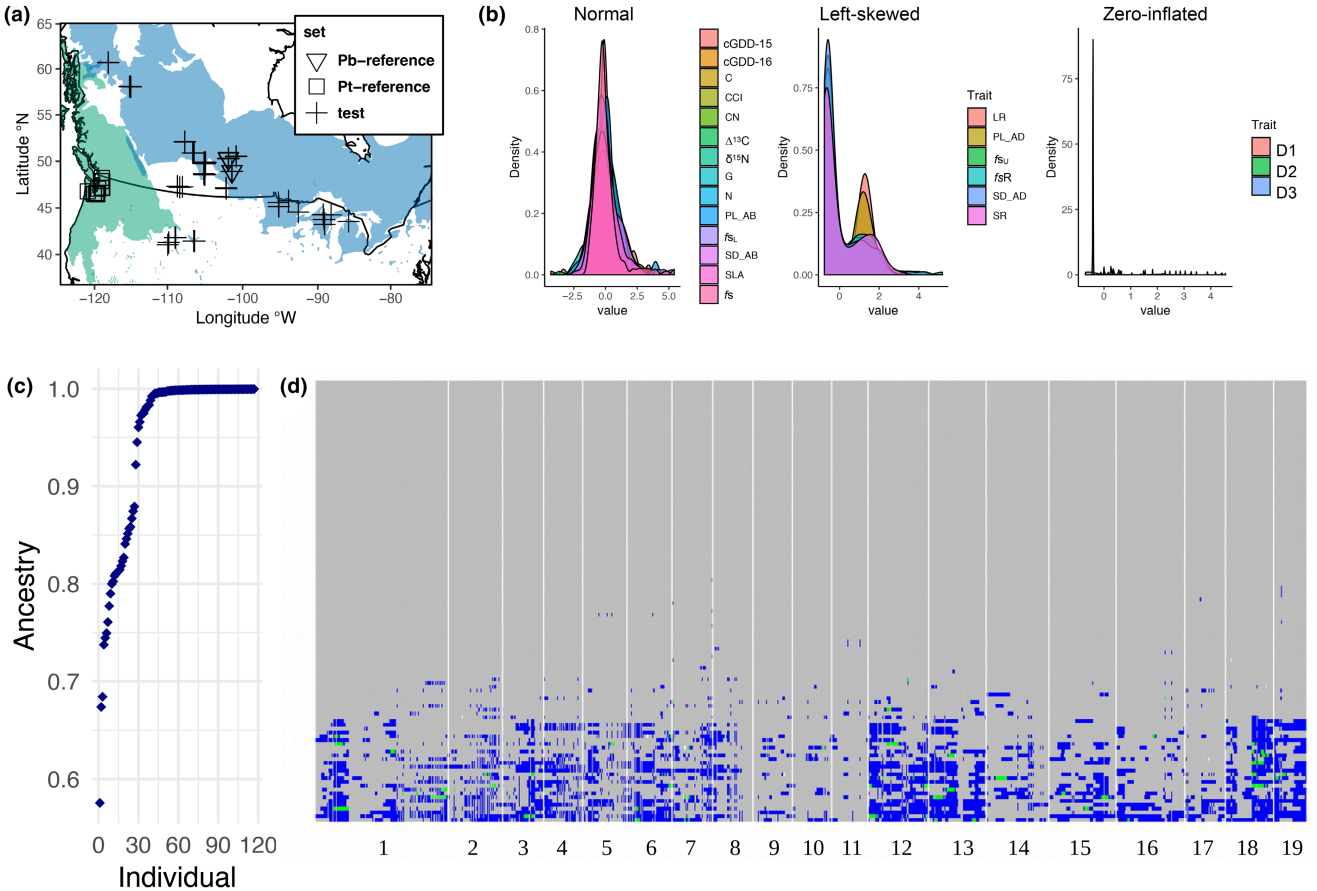
Map of collection localities in western North America with the range of *P. balsamifera* and *P. trichocarpa* colored in blue and green, respectively. Ranges from Little (1971) (a). Best linear unbiased predictors (BLUPs) from traits fall into three distribution categories: approximately normal, left‐skewed, and zero‐inflated (b). Global ancestry estimates from locus‐specific ancestries estimated by RASPberry for the admixture mapping test set (*N* = 117) (c). Local ancestry for each individual (in rows) and for every site (in columns) is colored as gray if the locus is homozygous for the *P. balsamifera* allele, blue for heterozygous loci, and green for homozygous for the *P. trichocrapa* allele (d).

Sequence filtering and merging of the combined 117 test individuals and 50 reference individuals yielded 31,523 SNPs for global and local ancestry estimation. Global ancestry estimates calculated from the local ancestries indicated we sampled a range of hybrid ancestries from unadmixed (RASPberry K2 q‐matrix = 0.9996) to admixed (RASPberry K2 q‐matrix 0.5754; Figure 1c). Based on previous analyses, the filial generation of the test set was known to include 18 P1.F_2_, 6 P1.P1F_1_, 10 P1.P1F_1_, and 83 unadmixed *P. balsamifera* (Fetter et al., 2021).

The locus‐specific ancestries revealed a patchwork of introgression loci, where 97.2% of loci were homozygous for the *balsamifera* ancestry allele (*N* = 3,417,375), 7.1% were heterozygous (*N* = 261,555), and only 0.2% (*N* = 8664) were homozygous for the *trichocarpa* ancestry alleles (Figure 1d). The predominance of heterozygous local ancestry sites was consistent with our expectations, given that the majority of samples are derived from advanced generation backcrosses into *balsamifera*.

### 3.2 Trait models

The traits we measured fall into three general patterns of distributions: normal, left‐skewed, and zero‐inflated (Figure 1b). Traits with normal distributions included the elemental and isotopic traits, bud flush, relative growth rate, and lower (abaxial) stomatal traits. Left‐skewed traits include the three stomatal ratio traits (SR, LR, *f*_*s*_R) and the upper (adaxial) stomatal traits which were left‐skewed as a result of many unadmixed *balsamifera* lacking upper stomata. The disease phenotypes were all zero‐inflated. After converting D2 into a binary disease presence/absence category, unadmixed *balsamifera* had 12 individuals with disease (9.8% of all *balsamifera*) and 110 without disease. Hybrids with *trichocarpa* ancestry had 19 individuals with disease (47.5%) and 21 individuals without disease sign (52.5%).

**FIGURE 2:**
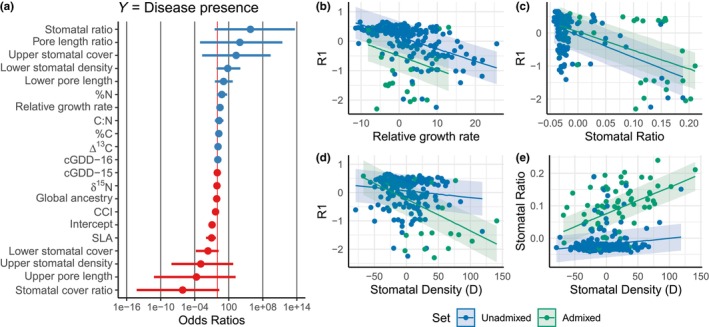
Forest plot of standardized regression coefficients from a logistic model with disease presence as the response. Blue and red points are positive and negative odds ratios, respectively. The red line is an odds ratio of 1. See Table S2 for logistic model output (a). Trade‐offs underlying disease resistance from random slope and intercept models using best linear unbiased predictors (BLUPs) as input (Table S3). Shaded areas indicate ± standard error of the slope (b–e).

We fit a logistic model of the presence/absence of disease to the 19 traits and global ancestry (Figure 2a, Table S2). Stomatal traits that were the sum of upper and lower traits were excluded from the logistic model. Stomatal ratio had the largest odds ratio (6.5e5), and the stomatal cover ratio had among the lowest (0.0), indicating variation of these traits contributed the most variance to the presence or absence of disease. Relative growth rate had a slight positive effect on disease presence (slope = 2.84). Ecophysiology and bud flush traits explained little variance of disease presence, although SLA had a significant negative effect (slope = 0.09, *p*‐value = .02).

Trade‐offs (i.e., negative slopes) between disease resistance and growth, stomatal ratio, and total stomatal density were recovered from random slope and intercept models of BLUPs with admixture status as a grouping effect (Figure 2b–d). In the growth‐resistance model, the intercepts between admixed and unadmixed individuals were offset by a value of 0.65, but the slopes were similarly negative for unadmixed (−0.41) or admixed (−0.46) sets The similar slopes indicate the decline of resistance as growth increases has a similar effect in both genetic backgrounds, but a substantial offset in the intercepts can be explained by the different genotypic variance between each parental species. In contrast, the intercepts and slopes for the SR‐resistance model (Figure 2c) were similar for both groups (Table S3), indicating the effect of adding more stomata to the upper leaf surface has a similar decay in resistance in both hybrid sets. Increased stomatal density has a nuanced effect on resistance, with only a slightly negative effect in unadmixed *balsamifera* (slope = −0.0023) but a significantly negative effect in *trichocarpa* hybrids (slope = −0.01 [−0.0157, −0.0067] 95% CI), suggesting the genotypic variance for this pair of traits is fundamentally different. The model for total stomatal density and SR demonstrates how stomata are deferentially apportioned, with admixed genotypes shifting more stomata to the upper surface in response to increased stomatal density (Figure 2e). Unadmixed *balsamifera* typically decrease the size of stomata to fit more on a leaf surface when density increases (linear model D~S slope = −0.1074***).

### 3.3 Admixture mapping

**FIGURE 3:**
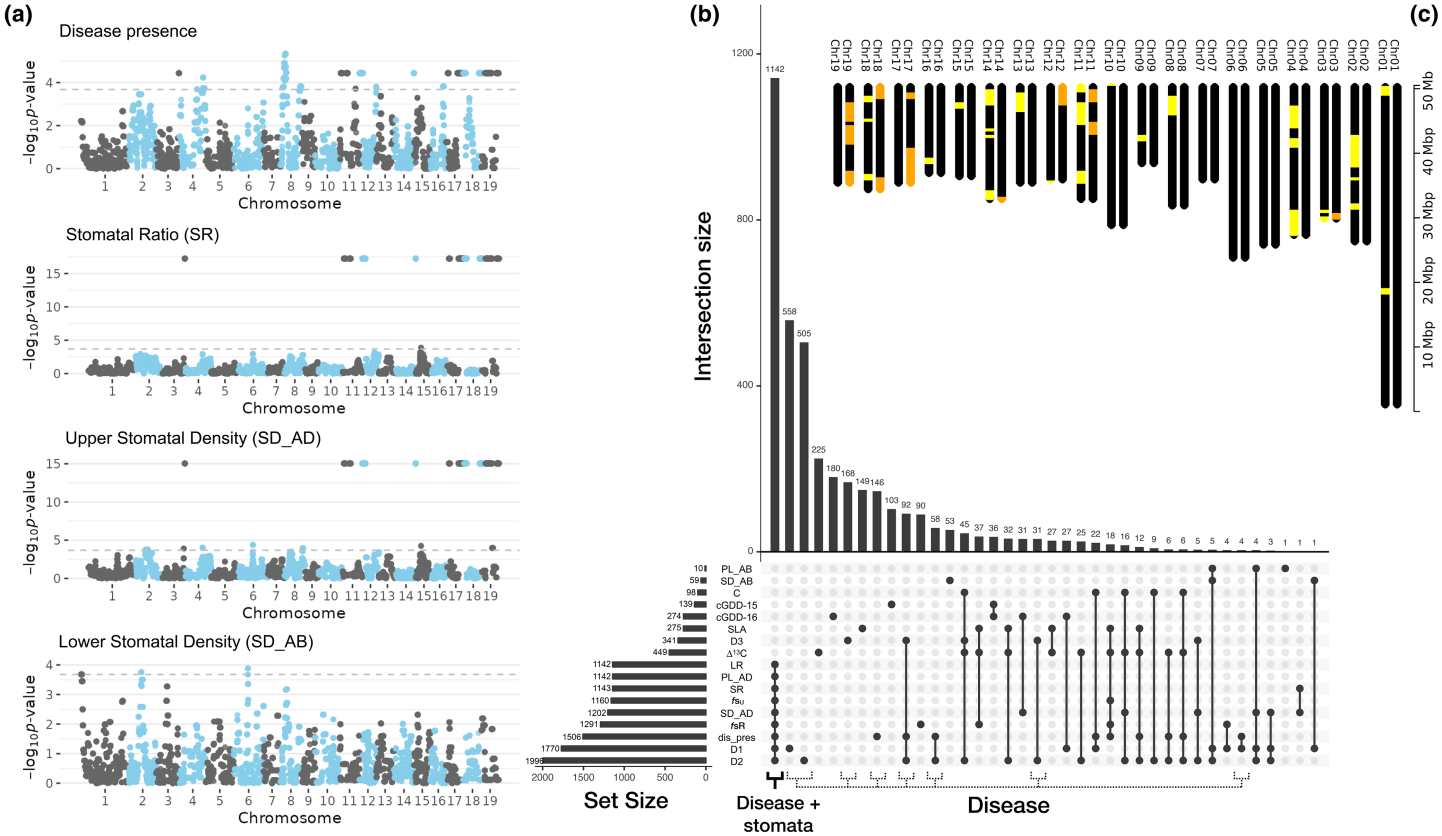
Results of admixture mapping genomic association tests. Manhattan plots for key traits. Cutoff value was α = .05/237.5 (gray dotted line) (a). UpSetR plots identified genes jointly associated with disease and stomatal trait variation (Disease + Stomata), or associated with disease, but independent of stomatal or ecophysiological traits (Disease). Overlap of gene lists from significant SNPs of each trait were found. The set size refers to the number of genes containing significant SNPs (b). Chromosomal locations of windows containing candidate genes for the disease and stomatal set (orange), and disease‐only set (yellow) are plotted (c).

Admixture mapping was performed with BMIX (Shriner et al., 2011) using the 31,523 locus‐specific ancestry estimates and 24 traits (Figure 3). Out of 746,928 tests, 3.2% (23,670 tests) had *p*‐values larger than the admixture burden corrected *p*‐value threshold (α = 0.05/237.50; Figure 3a and Figures S3–S5). Significant loci were contained among 13,997 genes, of which 28% (3877) were identified in only one test. Based on the evaluation of the Manhattan plots and the apparent co‐localization of significant loci to regions within chromosomes (Figure 3a), we clustered genes with UpSetR and revealed several groups of genes (Figure 3b). Notably, a group of genes was identified that were significantly associated in BMIX tests to D1, D2, disease presence/absence, *f*_*s*_R, SR, LR, and stomatal density and pore length (*N* = 1142). Another set of genes that only contained associations to disease traits independent of ecophysiology or stomatal traits was observed (*N* = 1562). These two sets of genes (disease plus stomata and disease‐only) mapped to locations on 7 and 13 chromosomes, respectively, out of the 19 total chromosomes in the *Populus* genome (Figure 3c). No significant loci were found for relative growth rate.

### 3.4 Selection scans

**FIGURE 4:**
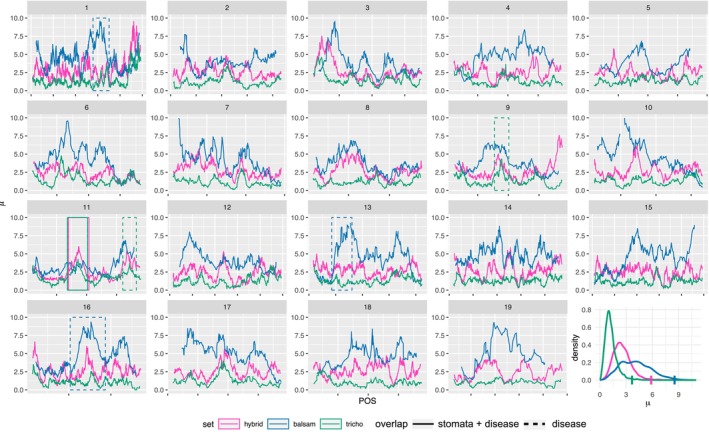
Results of RAiSD selection analysis (*μ* statistic) organized by chromosome. Each line color indicates a different set of individuals: hybrids (magenta), unadmixed *Populus balsamifera* (blue), and unadmixed *P. trichocarpa* (green). The overlap of Bayesian mixed model (BMIX) candidate genes and the top 1% of *μ* statistic outliers are indicated by the solid or dashed boxes for the disease plus stomata gene set, and disease‐only gene set, respectively. The density distribution of *μ*‐statistics is presented by set, and the vertical colored ticks on *x*‐axis indicate the top 1% of values for each set.

Positive selection was inferred using a sliding windows approach implemented with RAiSD (Alachiotis & Pavlidis, 2018). 31,545 SNPs were input into the selection scans of sliding windows of 50 SNPs in size yielded 13,395 windows in the hybrid set, 13,863 windows in the *P. trichocarpa* set, and 5559 windows in the western core *P. balsamifera* set. Each 50 SNP window varied in size in terms of actual nucleotides, with a median window size of 1.29e6 and a standard deviation of 3.7e5. The median value of the *μ* statistic for the *P. trichocarpa*, hybrid, and *P. balsamifera* data sets was 1.13, 2.42, and 3.97, respectively. *μ* statistics were plotted by window position (Figure 4). Nine chromosomal regions contained candidate genes from BMIX analyses that overlapped with the top 1% of *μ* statistic windows and contained 271 unique genes (Table 2, Figure 4).

**TABLE 2:** Summary of the number of genes contained within each chromosomal region containing both candidate genes from admixture mapping, and *μ* values in the top 1%.

BMIX gene set	*P. trichocarpa*	Hybrids	*P. balsamifera*
Stomata + disease	Chr11	Chr11	
	(46)	(46)	
Disease	Chr9, Chr11	Chr11	Chr1, Chr13, Chr16
	(2, 57)	(18)	(21, 12, 28)

*Note*: The number of genes found in each chromosomal region are given in parentheses.

### 3.5 Candidate genes and local ancestry class phenotypic distributions

**FIGURE 5:**
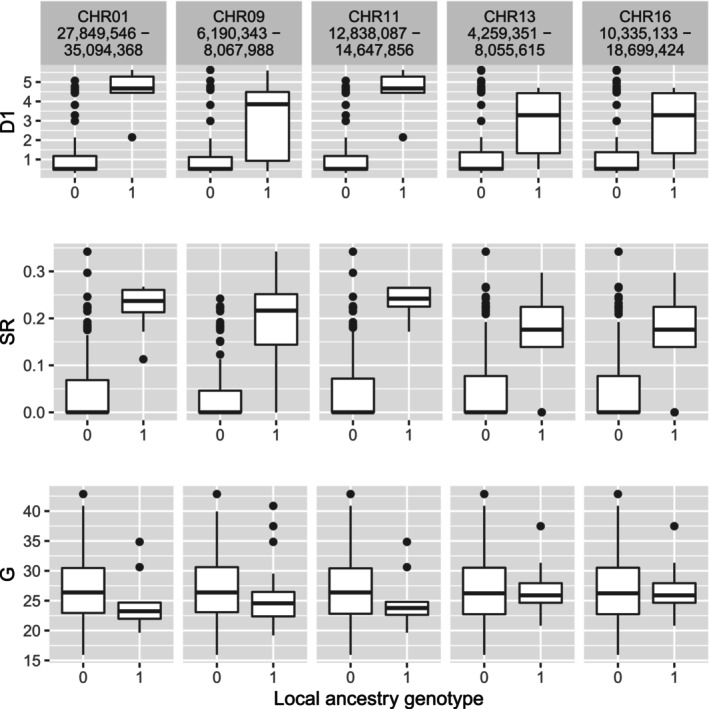
Allelic effects of polymorphic sites identified by admixture mapping and within the top 1% of selection scans. A shift in local ancestry genotype from homozygous for the *P. balsamifera* allele (0) to heterozygous (1) changes disease severity (D1), stomatal ratio (SR), and relative growth rate (G).

To further investigate the 271 genes we identified with BMIX that contained sites within the top 1% of selection windows, we determined the local ancestry sites that mapped to those genes and filtered them for monomorphic sites. We had 535 local ancestry sites that mapped to the 271 genes, and 221 of those sites were polymorphic. Local ancestry genotypes for SR, D1, and G were evaluated to remove sites that had either no phenotypes for the heterozygote local ancestry genotype (53 sites), or had only one individual in the heterozygote local ancestry genotype (5 sites). After filtering, 163 sites remained which shared one of five patterns of phenotypic distributions (Figure 5). Substituting a *P. trichocarpa* ancestry allele had a large average allelic effect for SR and D1, and to a lesser extent, a negative effect for G for sites on chromosome 1 and 11 (Table 3).

**TABLE 3:** Average allelic effects for substituting a *P. balsamifera* ancestry site (0) for *P. trichocarpa* (1).

Trait	Chr1	Chr9	Chr11	Chr13	Chr16
SR	1.87	1.61	2.00	1.17	1.17
D1	2.21	1.26	2.21	1.02	1.02
G	−1.88	−1.61	−2.00	−1.16	−1.16

*Note*: Phenotypes were scaled and standardized before calculating the allelic effect. The chromosomal coordinates for each region are provided in Figure 5.

We mapped the 163 sites to 99 genes and investigated each gene with popgenie.org (Sjödin et al., 2009), atgenie.org (Sundell et al., 2015), and TAIR (Berardini et al., 2015). We recorded information on gene family, function, and expression profiles that were relevant to disease or stomatal patterning. Among the 99 candidate genes, we identified genes involved in guard cell functioning, the immune system, detoxicants, lipid biosynthesis and trafficking, growth‐related proteins, cell wall production, abiotic/biotic stress response, epigenetic regulation, ubiquination, membrane transport, transcription factors, DNA replication, signal transduction, and floral development‐related genes (Table S5).

## 4 DISCUSSION

Hybrid zones can act as natural evolutionary experiments to observe the effects of shuffling genomic regions into novel genetic backgrounds (Chase et al., 2017; Christe et al., 2016; Fetter et al., 2021; Rieseberg et al., 2007; Suarez‐Gonzalez et al., 2018). Hybrid populations have increased genetic variance compared with parental species, allowing recombination to uncover genetic variants linked to traits. These populations offer unique opportunities to study the genetic basis of disease. Our genetic models showed a trade‐off between growth and disease resistance, influenced by stomatal traits, specifically the stomatal density ratio on upper and lower leaf surfaces. Using admixture mapping, we discovered candidate genes experiencing selection related to stomatal patterning, the immune system, and constitutive defenses. Candidate genes from multiple traits often mapped to the same genetic locus, possibly indicating pleiotropy or linked selection. Using selection scans, we identify positive selection as a dominant evolutionary force acting on genes underlying the trade‐offs. These findings provide insights into genomic mechanisms of trait evolution in response to selection from a foliar fungal pathogen.

### 4.1 Stomatal morphology and disease resistance

Stomatal morphologies have been demonstrated to evolve in response to a plant's growth strategy (McKown et al., 2019), growth form (Muir, 2015), or disease environment (Melotto et al., 2008). Simulation models indicate pathogens play an important role in determining the ratio of stomata on the upper to lower leaf surfaces (Muir, 2020). Using empirical disease and stomatal morphology data, we identified stomatal traits as being particularly important in explaining disease risk. Increasing stomatal density on the upper leaf surface was observed to correlate with increased growth in *P. trichocarpa* (McKown et al., 2019), while here, we observe a large increase in the risk of disease (log‐odds ratio = 6.5e5) with no benefit of increased growth (random slopes and intercepts output: y‐intercept for BxT = −0.44, 95% CI: [−2.3, 1.4]; slope = −0.05, 95% CI: [−0.08, −0.012]). When isolated from the numerous genetic effects of ancestry, stomatal ratio has a similar negative effect on resistance in both genetic groups (Figure 2d), supporting simulation models which were conducted in the absence of genetic architecture (Muir, 2020).

Interestingly, the effect of increasing total stomatal density on resistance is not the same between hybrid sets (see slopes in Figure 2d). *P. balsamifera* genotypes tend to pack stomata more tightly on the lower leaf surface with increased density, rather than shifting stomata to the upper leaf surface in *P. trichocarpa* hybrids (Figure 2e). Contrasting stomatal‐growth‐defense trait syndromes are a feature of poplars species, and these models, focused on a single species pair, reiterate complementary analyses of naturally formed hybrids with *P. angustifolia* and *P. deltoides* (Fetter et al., 2021). Trade‐offs observed in hybrid and non‐hybrid populations indicate stomatal traits are constantly shifting in response to the underlying genotypic variance and biotic environment, a finding in support of our previous work.

### 4.2 Growth–resistance trade‐off

We found a trade‐off between disease resistance and growth, stomatal ratio, and stomatal density that differed between unadmixed and admixed poplars. Trade‐offs have been well‐studied in the evolutionary literature as they indicate physiological limits to adaptation, and are responsive to ecological and environmental contexts (Cope et al., 2021). The admixture mapping results that indicate the genetic structure of the trade‐offs are highly polygenic. Simulation studies indicate polygenic traits under phenotypic selection have higher evolutionary rates than traits with large‐effect loci (Kardos & Luikart, 2021). The positive selection we identified tends to support the hypothesis that allele frequencies underlying ecologically important polygenic traits experience positive selection and are capable of rapid evolution. These data support the growing consensus that many traits important for adaptation in natural environments are polygenic (Bomblies & Peichel, 2022).

### 4.3 Advanced generation hybrids and selection against *trichocarpa* ancestry

We collected a sample from across the southern and western range of *P. balsamifera* with the intent of collecting unadmixed *P. balsamifera* genotypes. However, the prevalence of hybrid zones in *Populus* and sampling dormant cuttings increases the likelihood of collecting hybrids. Genotyping these accessions revealed the tail of a hybrid ancestry distribution across a geographically large region. Backcrossing was observed in the sample, with hybrid ancestry starting at 0.57 and increasing (Figure 1). In a genetic landscape of hybrids, introgression can be expected to occur and has been demonstrated to underlie important ecological adaptations in poplars. Introgression of an 880‐kb genomic region on chromosome 15 from *P. balsamifera* into *P. trichocarpa* was demonstrated to confer increased ecological differentiation, perhaps allowing genotypes with the introgressed region to inhabit climatically challenging sites (Suarez‐Gonzalez et al., 2016). In a *P. trichocarpa*, *P. angustifolia*, and *P. balsamifera* trihybrid zone, introgression of soil ion detoxification and photoperiod regulation genes was observed (Chhatre et al., 2018).

Given the documented importance of adaptive introgression in *Populus*, we expected to find genes from *P. tricochrpa* conferring an adaptive advantage in our sample. However, we found little evidence of a fitness component advantage in admixed genotypes with genomic blocks of *trichocarpa* ancestry. Mixed‐effect models demonstrated *trichocarpa* hybrids had lower disease resistance overall (see intercept of Figure 2b). Additionally, the disease cost of increasing stomatal density was higher in *trichocarpa* hybrids (see slope of Figure 2d), further indicating global *trichocarpa* ancestry was selected against. At a finer scale, admixture mapping identified five chromosomal regions significantly associated with decreased disease resistance that are also associated with decreased growth and increased stomatal ratio (Figure 5). In all five of these chromosomal regions, the heterozygote has decreased resistance and growth, indicating they are being selected against. These data generally suggest hybrid breakdown in novel disease communities occurs, and limits introgression and gene flow between these species. Hybrid breakdown has been previously reported in *Populus alba* × *treumla* and invoked to explain reproductive isolation between species (Christe et al., 2016). These data suggest negative selection will act to protect a species' genome from introgression in response to increased mortality from disease.

### 4.4 Genomic basis of disease resistance

Admixture mapping revealed a complex, polygenic basis of disease resistance. While the mixed models used here rely on changes in locus‐specific ancestry, indels may also contribute effects to phenotypic variation through modifications of codons and amino acid sequences in proteins, modified sequences of regulatory regions (e.g., binding specificity in promoters), or through other means. The shape of *p*‐value distributions can vary widely between traits, and their distributions are ultimately due to the underlying test statistics influenced by (1) the strength of the actual association, (2) patterns of linkage disequilibrium between a causal locus and the genotyped locus (which are likely to differ), and (3) patterns of neutral variation driven by mutation and drift near a locus. Here, we potentially observe all three phenomena influencing the distribution of *p*‐values. For example, in many of the tests, including non‐significant tests, *p*‐values are correlated at sites on chromosomes 3, 11, 12, 14, and 17 to 19. These blocks of sites are likely in complete linkage. In contrast, some *p*‐value distributions contain relatively few significant loci, for example, lower stomatal density, which may reflect the difficulty of the mixed models to account for mutation or drift governing allele frequencies near associated sites. Interestingly, no significant loci were detected for the relative growth rate, likely indicating it is a polygenic trait with many loci of small effect.

Putatively associated SNPs lie near candidate genes that fell into broad categories related to stomatal function, the plant immune system, constitutive defenses, and growth regulators. While some Pfam descriptions from genes associated with disease resistance are obviously involved in defense (e.g., LRR‐N terminal domain), others are not and indicate a plant's overall physiology contributes to resistance.

We identified several genes with known functions for stomatal guard cell regulation. Guard cells open and close the aperture pore of a stoma via reversible changes in the concentration of ions, subsequently altering cellular turgor pressure. Reactive oxygen species (ROS) and calcium ions can function as messenger molecules in stomatal signaling pathways and can be pumped into guard cells to change the ionization of the cell (Lecourieux et al., 2006). Among our candidates are an ion transmembrane transporter (Potri.016G115500) and an ROS‐mediated signal transduction protein (Potri.011G112700).

Plant defenses against pathogens include both constitutive and induced defenses. A successful host‐induced immune response is initiated by recognizing the presence of pathogen‐associated molecular patterns (PAMPs). PAMP‐triggered immunity (PTI) can be induced by the detection of PAMPs, which results in a signaling cascade to initiate a broad‐spectrum defensive response by the host plant (Jones & Dangl, 2006). We detected candidate genes for the plant immune system, including signal transduction proteins (Potri.011G116200), LRR proteins (Potri.011G116900), oxidative stress detoxifying proteins (Potri.011G113000), and a negative regulator of pathogenesis responsive genes (Potri.011G121200). Constitutive defenses can include morphological or chemical defenses that limit colonization or growth of a pathogen. The plant cuticle is composed of lipids which can limit colonization of pathogens on a leaf surface. Differences in cuticle lipid chemistry have been linked to variation of *Melampsora* infection in *P. trichocarpa* (Gonzales‐Vigil et al., 2017). We observed six candidate genes involved in lipid biosynthesis or transport (Potri.001G317400, Potri.016G115800, Potri.016G116400, Potri.016G113800, Potri.016G118000, and Potri.001G316600), and two candidate genes involved in cell wall homeostasis (Potri.013G056800 and Potri.016G114300).

Finally, we identified several candidate genes involved in transcriptional regulation of growth (Potri.011G115400), cell boundary specification (Potri.011G121300), and an auxin transmembrane transporter (Potri.016G113600) which may potentially lie at the intersection of growth–defense trade‐offs.

## 5 CONCLUSION

We used admixture mapping to identify genes under selection that are associated with disease severity to a fungal pathogen. These results provide evidence that admixture mapping can be used to find ecologically relevant genes and supports the hypothesis that variation of loci within genes can have effects that cascade to ecological relationships and the broader environment (Wymore et al., 2011). In this study, hybrid genotypes serve as a reservoir of disease, an observation shared by other studies in poplars (Whitham, 1989). The shared signals of genomic association between disease and stomatal patterning likely indicate stomatal traits are under strong positive selection, in particular, the stomatal ratio. These results suggest stomata are an important component of an integrated physiological regulation of growth and defense in wild plants.

## AUTHOR CONTRIBUTIONS

**Karl C. Fetter:** Data curation (equal); formal analysis (lead); investigation (lead); methodology (equal); writing – original draft (lead); writing – review and editing (equal). **Stephen R. Keller:** Conceptualization (lead); data curation (lead); funding acquisition (lead); project administration (lead); resources (lead); supervision (lead); writing – original draft (supporting); writing – review and editing (equal).

## CONFLICT OF INTEREST STATEMENT

The authors have no conflict of interest to declare.

## Supporting information


Data S1:

## Data Availability

Raw sequence reads are available for download at NCBI SRA (SRP070954). Phenotypes from the common garden are available as supporting information in Fetter et al. (2021). Cuticle micrographs are deposited on Dryad (doi: 10.5061/dryad.kh2gv5f).
