# Supplementary material for: Admixture mapping and selection scans identify genomic regions associated with stomatal patterning and disease resistance in hybrid poplars
Source: Ecol Evol. 2023 Oct 24;13(10):e10579. doi: 10.1002/ece3.10579 (PMC10597741; doi:10.1002/ece3.10579)
Supplement: Supplementary file 1 — Data S1: [file ECE3-13-e10579-s001.pdf]

Supporting Information

Table S1: Population locality and sample size summaries for individuals used in admixture mapping. Unadmixed *Populus balsamifera* (ref-*Pb.*, N = 25) and *P. trichocarpa* (ref-*Pt.*, N = 25) were selected to generate locus-specific ancestries for admixture mapping in the test set (N = 117).

Pop.	Set	N	Lat.	Lon.
BESC	ref-*Pt.*	15	47.81	-122.01
GW	ref-*Pt.*	9	47.30	-122.58
Nisqually	ref-*Pt.*	1	47.03	-122.67
DCK	ref-*Pb.*	20	51.60	-101.73
CLK	test	6	54.22	-110.08
HBY	ref-*Pb.*/test	23	52.89	-102.39
CYH	test	4	49.64	-109.98
FNO	test	8	58.50	-122.37
JKH	test	6	43.83	-110.47
MMT	test	6	49.88	-102.59
MSG	test	4	44.31	-106.90
OFR	test	14	53.14	-101.10
OUT	test	4	51.14	-106.20
SKN	test	4	52.33	-106.27
SSR	test	12	44.46	-109.61
TUR	test	7	53.20	-108.32
USDA12	test	6	46.58	-88.030
USDA13	test	2	46.08	-88.030
USDA14	test	1	45.58	-88.030
USDA15	test	1	46.42	-86.870
USDA18	test	1	45.42	-84.500
USDA3	test	1	47.17	-91.670
USDA7	test	3	48.42	-92.980
USDA8	test	1	48.33	-94.520
USDA9	test	4	47.92	-94.520
WLK	test	4	60.05	-128.44
Total		167		

Table S2: Output of logistic model of disease presence and traits.

Term	Estimate	Std.Error	P.Value	P.Stars
SR	656951.99	8.07	0.10	
LR	8966.05	8.13	0.26	
f_{S_U}	1978.26	6.72	0.26	
SD_AB	65.20	2.36	0.08	
PL_AB	12.05	1.76	0.16	
N	5.91	1.03	0.08	
G	2.84	0.59	0.08	
CN	1.93	0.84	0.43	
C	1.77	0.62	0.36	
D13C	1.28	0.48	0.61	
bf16	1.17	0.45	0.73	
bf15	0.94	0.51	0.91	
d15N	0.82	0.43	0.65	
global_ancestry	0.75	0.61	0.63	
CCI	0.47	0.68	0.26	
(Intercept)	0.11	0.53	0.00	***
SLA	0.09	1.01	0.02	*
f_{S_L}	0.02	2.28	0.09	
SD_AD	0.00	5.87	0.25	
PL_AD	0.00	8.02	0.29	
PR	0.00	8.25	0.09	

Table S3: Model output from random intercept and slope models. Models were fit in brms with BLUPs as input data. See Fig. 2 for plots of each model. Abbreviations: R1 = disease resistance (2015); G = relative growth rate; SR = stomatal ratio; D = log total stomatal density. Ancestry was either BxB or BxT.

R1∼ (1 + G | ancestry)

	Estimate	Est.Error	Q2.5	Q97.5
Intercept				
BxB	0.2067035	0.8917482	-1.683928	2.118894
BxT	-0.4433149	0.8964821	-2.345828	1.458727
Slope				
	Estimate	Est.Error	Q2.5	Q97.5
BxB	-0.04135374	0.005367762	-0.05190709	-0.0307486
BxT	-0.04623021	0.017100377	-0.07899603	-0.0118752

R1∼ (1 + SR | ancestry)

	Estimate	Est.Error	Q2.5	Q97.5
Intercept				
BxB	-0.2606446	0.8399156	-2.184307	1.321452
BxT	-0.1555295	0.8647422	-2.212310	1.475180
Slope				
	Estimate	Est.Error	Q2.5	Q97.5
BxB	-6.695734	1.109421	-8.832040	-4.505172
BxT	-5.643501	1.103754	-7.966181	-3.590200

R1∼ (1 + D | ancestry)

	Estimate	Est.Error	Q2.5	Q97.5
Intercept				
BxB	0.03483479	0.6257267	-1.490817	1.312665
BxT	-0.25450876	0.6343904	-1.808712	1.010025
Slope				
	Estimate	Est.Error	Q2.5	Q97.5
BxB	-0.002386611	0.001398987	-0.005101953	0.0003786823
BxT	-0.011194649	0.002285893	-0.015724202	-0.0066023706

SR∼ (1 + D | ancestry)

	Estimate	Est.Error	Q2.5	Q97.5
Intercept				
BxB	0.4296254	0.9106484	-0.7843780	1.948828
BxT	0.5240732	0.9123055	-0.6918405	2.049142
Slope				
	Estimate	Est.Error	Q2.5	Q97.5
BxB	0.0001817294	8.283463e-05	1.031871e-06	0.0003163581
BxT	0.0008086642	1.521083e-04	5.980606e-04	0.0010819346

Table S4: Summary of populations used in the RAiSD selection scan analysis.

Pop.	Set	N	Lat.	Lon.
CLK	*P. balsamifera*	12	54.22	-110.08
DCK	*P. balsamifera*	12	51.60	-101.73
HBY	*P. balsamifera*	6	52.90	-102.39
MMT	*P. balsamifera*	13	49.88	-102.59
OFR	*P. balsamifera*	12	53.14	-101.10
OUT	*P. balsamifera*	5	51.15	-106.26
SKN	*P. balsamifera*	10	52.35	-106.64
TUR	*P. balsamifera*	10	53.20	-108.36
CYH	Hybrid	4	49.64	-109.98
FNO	Hybrid	8	58.51	-122.38
HBY	Hybrid	1	52.93	-102.39
JKH	Hybrid	6	43.84	-110.47
MSG	Hybrid	4	44.32	-106.91
SKN	Hybrid	1	52.22	-106.27
SSR	Hybrid	7	44.46	-109.60
USDA12	Hybrid	3	46.58	-88.03
USDA18	Hybrid	1	45.42	-84.50
WLK	Hybrid	4	60.05	-128.44
13	*P. trichocarpa*	2	54.15	-128.60
31	*P. trichocarpa*	8	49.71	-125.06
Nisqually	*P. trichocarpa*	9	47.10	-122.64
Nooksack	*P. trichocarpa*	8	48.80	-122.17
Olympic Penninsula	*P. trichocarpa*	3	47.59	-122.90
Puyallup	*P. trichocarpa*	5	47.09	-122.20
Skagit	*P. trichocarpa*	5	48.51	-122.07
Skykomish	*P. trichocarpa*	6	47.82	-121.86
Total		165		

Table S5: Descriptions of candidate genes. *Arabidopsis* orthologs were identified by the best BLAST hit from www.popgenie.org or through BLAST results from The Arabidopsis Information Resource (www.arabidopsis.org). See text for details regarding candidate gene selection.

Potri ID	*At*-ortholog	Pfam description
Guard cell function		
001G317800	AT1G15690	NA
001G318800	AT4G30990	Down-regulated in metastasis
011G112700	AT3G14420	oxidoreductase activity
013G057500	AT5G56540	NA
016G115500	AT3G51860	transmembrane transport
016G117200	AT3G51850	Protein tyrosine kinase, protein phosphorylation EF hand
Immune system & detoxicants		
011G113000	AT1G78380	protein binding, Glutathione S-transferase, C-terminal domain
011G116200	AT4G27540	PRA1 family protein
011G116900	AT5G53890	Leucine rich repeat N-terminal domain
011G117100	AT3G23560	MatE (multi antimicrobial extrusion protein)
011G117200	AT3G23550	MatE (multi antimicrobial extrusion protein)
011G117300	AT3G23560	MatE (multi antimicrobial extrusion protein)
011G117400	AT3G23550	MatE (multi antimicrobial extrusion protein)
011G118200	AT1G28280	VQ motif
011G118900	AT3G15353	NA
011G121200	AT4G12560	F-box associated, protein binding
013G058500	AT3G03960	cellular protein metabolic process
016G118100	AT3G51830	SacI homology domain
Lipid biosyntheis & transport		
001G316600	AT3G05180	NA
001G317400	AT4G22330	ceramide metabolic process
016G113800	AT1G74720	protein binding
016G115800	AT2G38180	lipid metabolic process
016G116400	AT5G01410	pyridoxal phosphate biosynthetic process
016G118000	AT3G51840	acyl-CoA dehydrogenase activity
Growth related		
011G121300	AT5G17260	no apical meristem (NAM) protein
011G115400	AT5G53950	regulation of transcription, DNA-dependent
016G113600	AT2G38120	transmembrane amino acid transporter protein
016G114600	AT5G01270	double-stranded RNA binding
016G118400	AT4G33270	WD domain, G-beta
Cell wall related		
013G056800	AT5G19780	Tubulin/FtsZ family, GTPase domain
016G114300	AT2G20340	carboxylic acid metabolic process
Abiotic/biotic stress responsive		
001G316900	AT4G04980	NA
001G317000	AT4G04980	NA
001G317300	AT5G49210	NA
001G318900	NA	NA
011G115200	AT3G30390	NA
013G056900	AT5G18100	superoxide metabolic process
013G057700	AT3G03890	FMN binding
Epigenetics & DNA replication		
001G316200	AT3G01320	nucleus, Histone deacetylase (HDAC) interacting
001G316300	AT3G01320	nucleus
001G316500	AT1g04840	PPR repeat
001G317500	AT4G13780	tRNA binding, aminoacyl-tRNA ligase activity

Continued on next page

Table S5 – continued from previous page

Potri ID	At-ortholog	Pfam description
001G317700	AT2G31740	methyltransferase activity
009G085400	AT1G44910	protein binding, FF domain
011G114100	AT1G17160	pfkB family carbohydrate kinase
011G116000	AT4G13870	nucleobase, nucleoside, nucleotide and nucleic acid metabolic process
011G116100	AT5G53920	protein methyltransferase activity
013G056700	AT5G19790	apetela 2 domain (AP2 domain) transcription factor
013G057000	AT5G18110	translation initiation factor activity
013G058800	AT1G54390	protein binding
013G058900	AT4G13650	PPR repeat
016G116900	AT5G05610	PHD-finger
016G117300	At5g01380	Myb/SANT-like DNA-binding domain
Ubiquination		
001G316400	AT1G04850	protein binding, PUB domain
011G112800	AT3G14400	ubiquitin thiolesterase activity
016G115300	AT5G01520	zinc finger, C3HC4 type (RING finger)
Flower related		
001G316800	AT1G04910	GDP-fucose protein O-fucosyltransferase
011G112500	AT1G31660	bystin
011G115000	AT5G57850	catalytic activity
016G116300	AT5G01450	NA
016G117400	AT5G01370	NA
Membrane transporters		
001G316700	NA	Rab GTPase activator activity
001G317100	AT4G13750	NA
001G317200	AT4G13750	NA
001G318700	AT1G71900	NA
016G115400	AT5G01500	Mitochondrial carrier protein
Secondary metabolism related		
001G317600	AT1G04920	sucrose metabolic process
016G115600	AT2G25300	NA
Signal transduction		
011G114200	NA	protein transport, Plug domain of Sec61p
016G114800	AT3G09010	protein phosphorylation
016G119300	AT2G38280	purine ribonucleoside monophosphate biosynthetic process
Unknown genes, functions, or enigmatic		
001G317900	NA	NA
001G318000	NA	NA
009G085500	AT2G20240	NA
011G112600	NA	NA
011G115100	AT5G53970	transferase activity, transferring nitrogenous groups
011G115300	NA	Plant mobile domain
011G115900	NA	NA
011G117000	NA	NA
011G118000	AT5G53860	NA
011G118100	NA	metal ion binding
013G057100	AT3G03860	cell redox homeostasis
013G057200	AT5G18130	NA
013G058600	AT1G64770	NA
016G113900	NA	NA
016G114000	NA	NA
016G114100	NA	NA
016G114700	AT2G40060	clathrin coat of trans-Golgi network vesicle
016G115700	NA	NA
016G116200	AT5G01460	NA
016G116800	NA	NA
016G117900	NA	NA

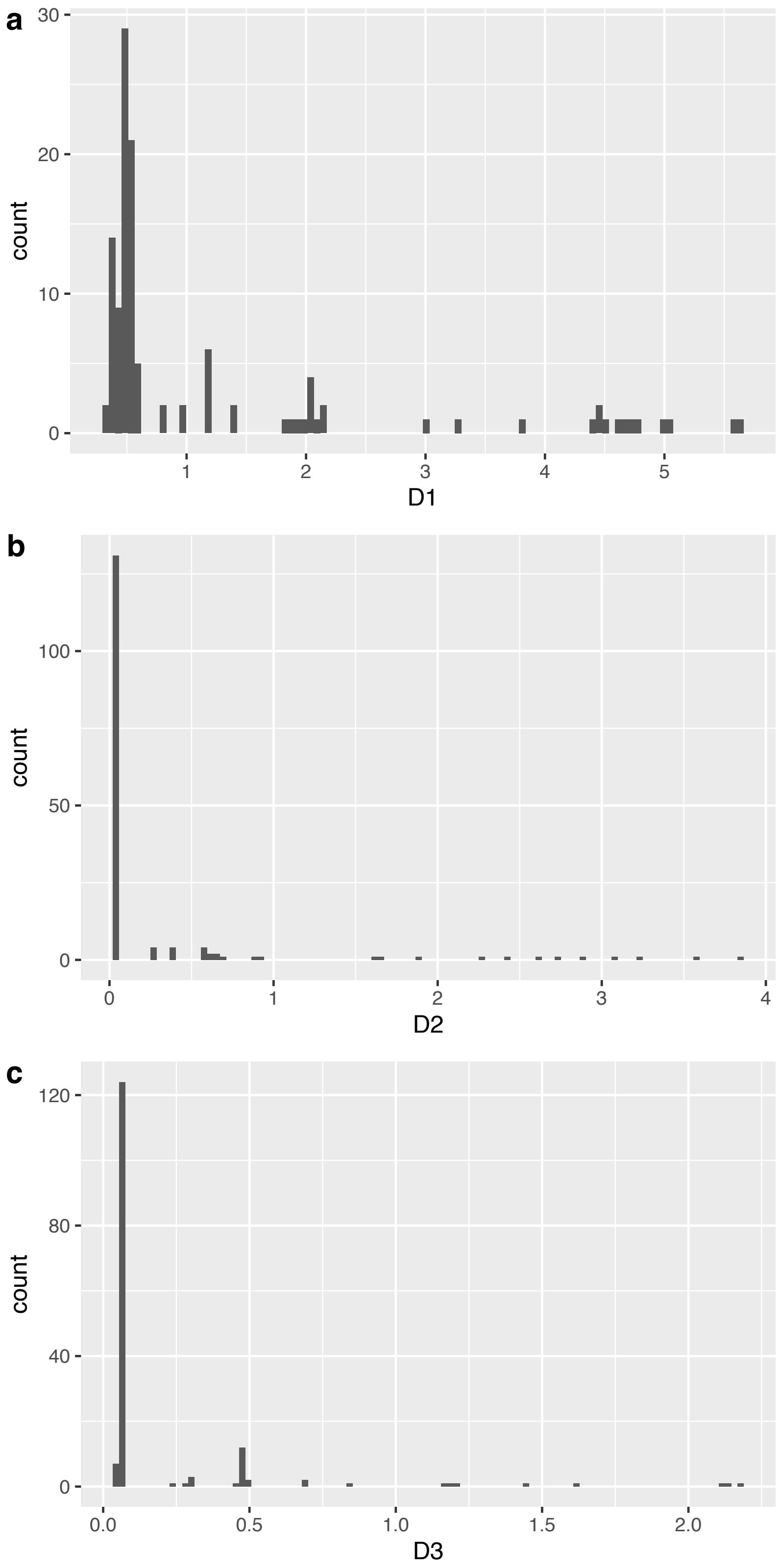

Figure S1: Disease severity BLUPs. The binary disease presence/absence response was converted from D2 (panel b).

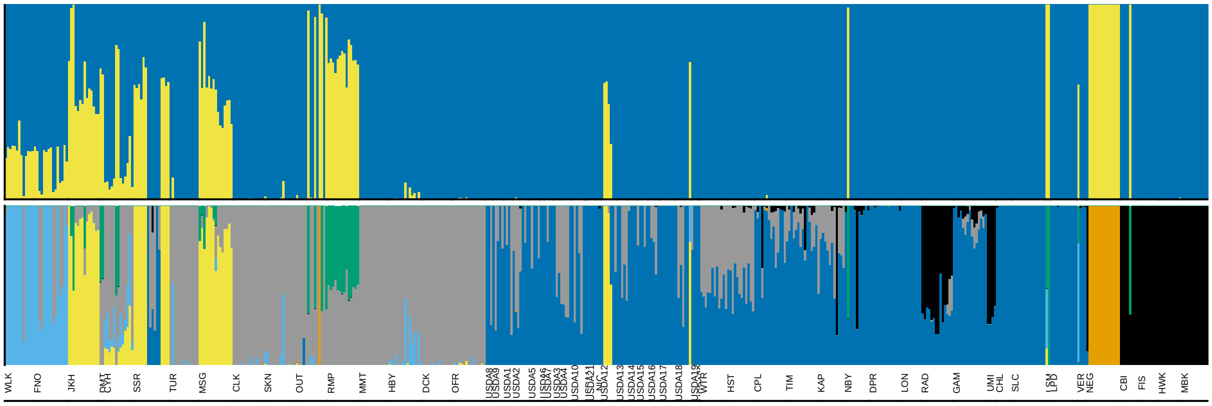

Figure S2: Global ancestry estimated from ADMIXTURE (Alexander *et al.*, 2009) at K = 2 (top) and K = 7 (bottom).

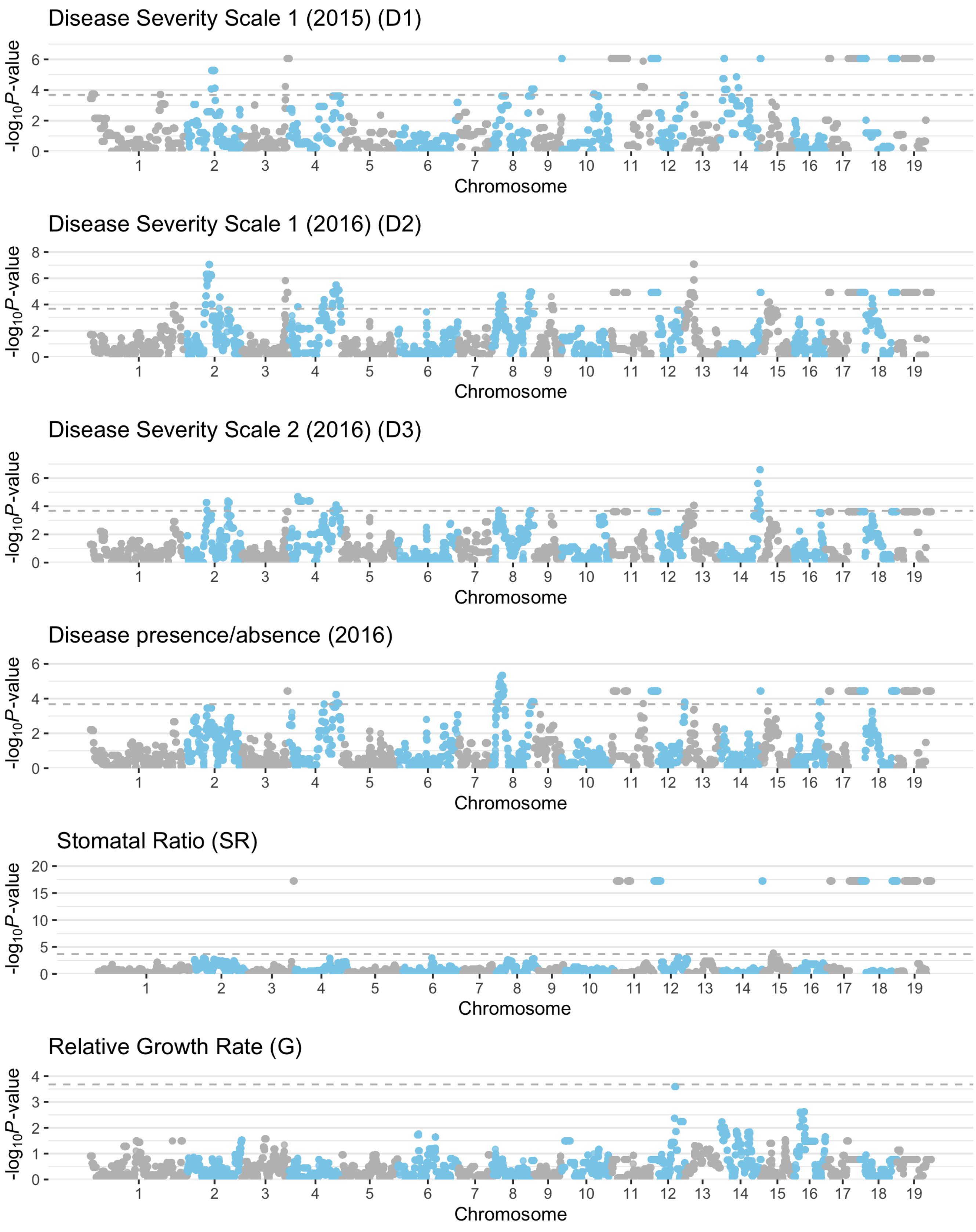

Figure S3: Individual manhattan plots of p-values from BMIX tests. See Table 1 for trait abbreviation definitions.

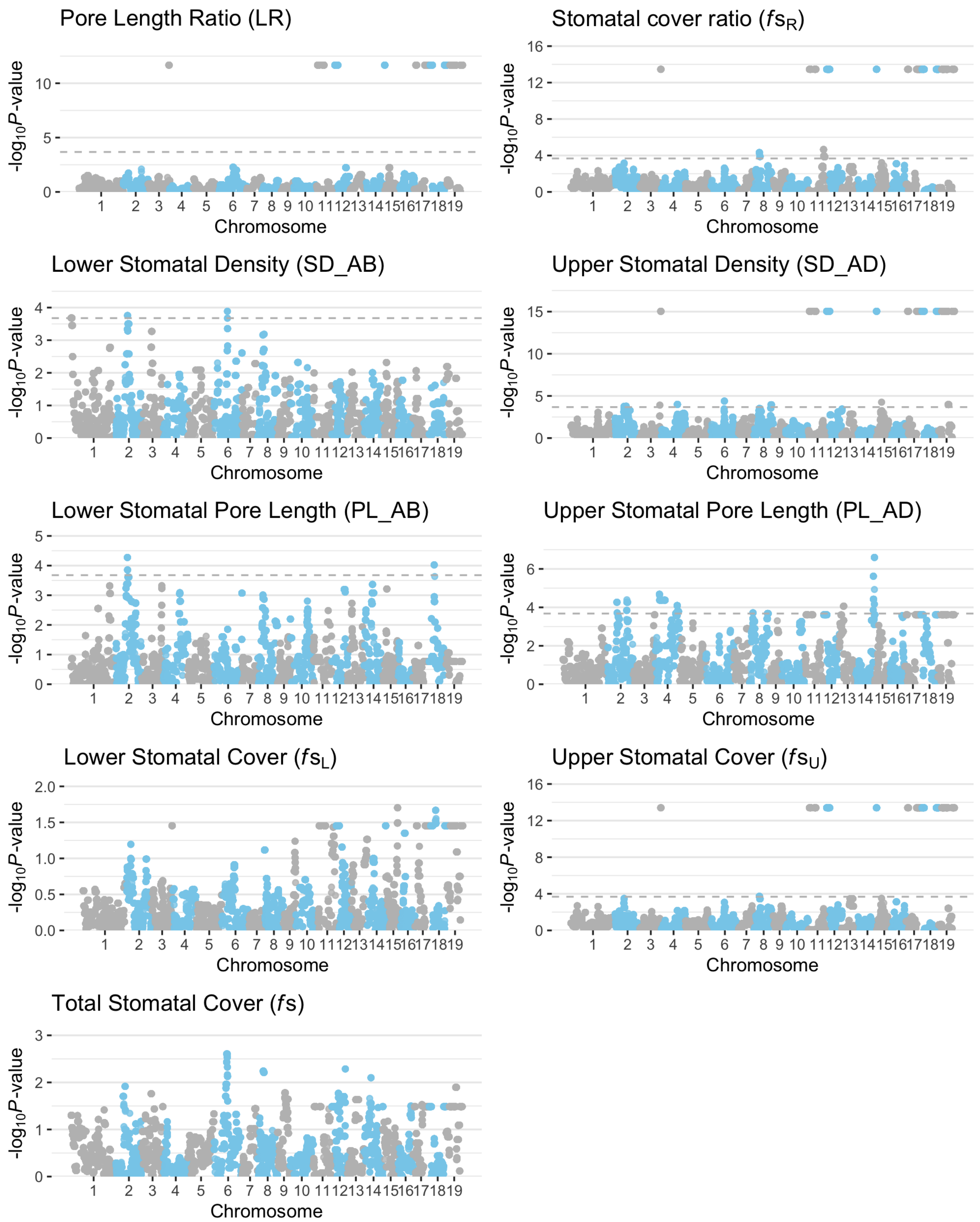

Figure S4: Individual manhattan plots of p-values from BMIX tests. See Table 1 for trait abbreviation definitions.

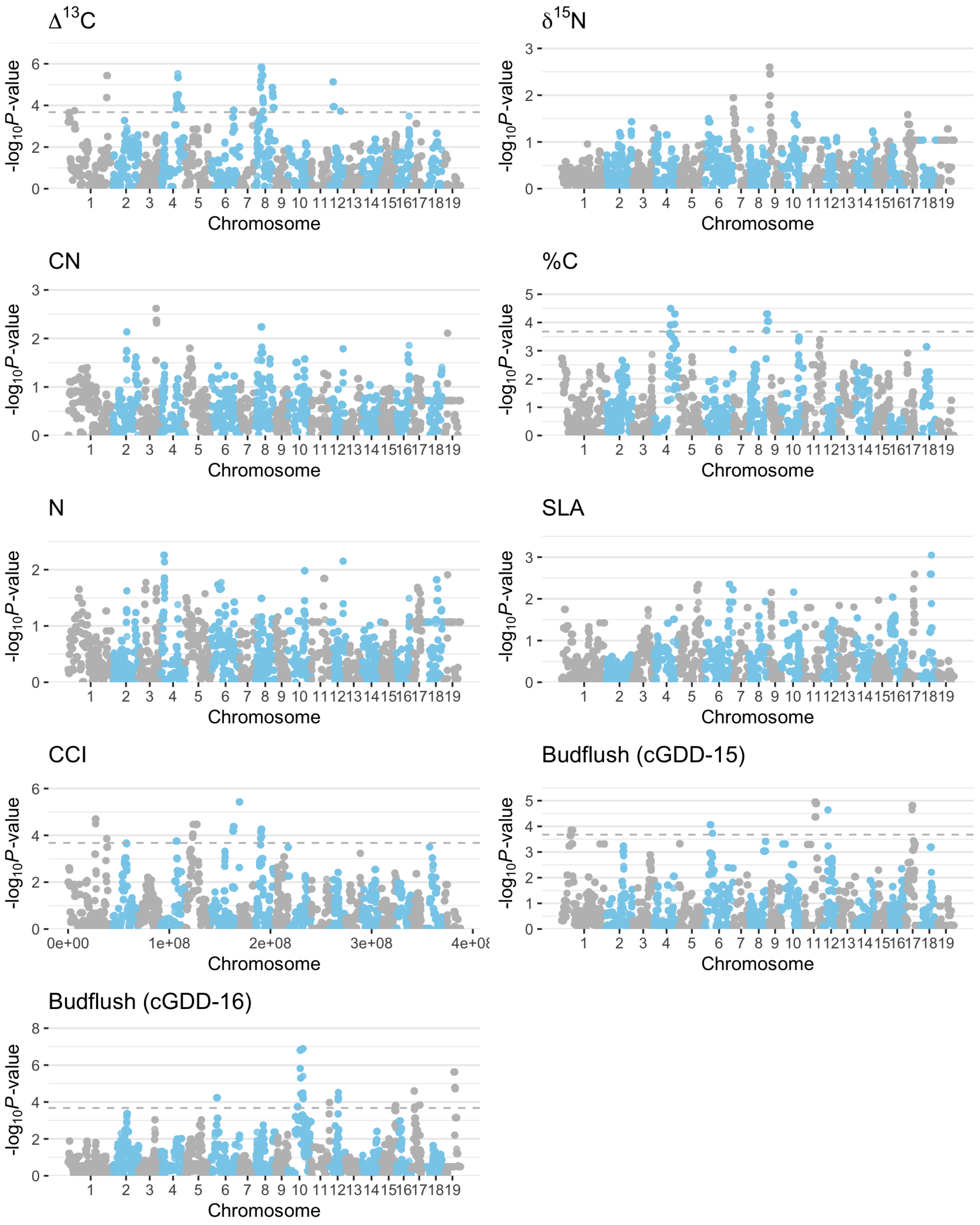

Figure S5: Individual manhattan plots of p-values from BMIX tests. See Table 1 for trait abbreviation definitions.
